# Performance comparison of machine learning techniques in sleep scoring based on wavelet features and neighboring component analysis

**DOI:** 10.7717/peerj.5247

**Published:** 2018-07-25

**Authors:** Behrouz Alizadeh Savareh, Azadeh Bashiri, Ali Behmanesh, Gholam Hossein Meftahi, Boshra Hatef

**Affiliations:** 1 Student Research Committee, School of Allied Medical Sciences, Shahid Beheshti University of Medical Scinces, Tehran, Iran; 2 Health Information Management Department, School of Allied Medical Sciences, Tehran University of Medical Sciences, Tehran, Iran; 3 Student Research Committee, School of Health Management and Information Sciences Branch, Iran University of Medical Sciences, Tehran, Iran; 4 Neuroscience Research Center, Baqiyatallah University of Medical Sciences, Tehran, Iran

**Keywords:** Sleep scoring, Artificial neural network, Neighboring component analysis, Machine learning, Support vector machine, Wavelet tree analysis

## Abstract

**Introduction:**

Sleep scoring is an important step in the treatment of sleep disorders. Manual annotation of sleep stages is time-consuming and experience-relevant and, therefore, needs to be done using machine learning techniques.

**Methods:**

Sleep-EDF polysomnography was used in this study as a dataset. Support vector machines and artificial neural network performance were compared in sleep scoring using wavelet tree features and neighborhood component analysis.

**Results:**

Neighboring component analysis as a combination of linear and non-linear feature selection method had a substantial role in feature dimension reduction. Artificial neural network and support vector machine achieved 90.30% and 89.93% accuracy, respectively.

**Discussion and Conclusion:**

Similar to the state of the art performance, the introduced method in the present study achieved an acceptable performance in sleep scoring. Furthermore, its performance can be enhanced using a technique combined with other techniques in feature generation and dimension reduction. It is hoped that, in the future, intelligent techniques can be used in the process of diagnosing and treating sleep disorders.

## Introduction

Sleep is a behavioral state characterized by the lack of interaction between an individual and the environment as well as a relative motor quiescence (Nofzinger et al., 1997). It is worth mentioning that the undeniable impact that sleep has on various human physical and mental activities make it a significant factor in human health (Hays & Stewart, 1992; Czeisler & Klerman, 1999; Tibbitts, 2008; Tavallaie et al., 2005). Thus, it is clear that sleep disorders can lead to devastating effects on various aspects of human life (Buysse et al., 2010).

In regard to the treatment of sleep disorders, polysomnography (PSG) can be considered as the main tool for collecting as well as measuring the electrophysiological signals to analyze body functions during sleep (Nofzinger, 2005). Therefore, an important step here would be hypnogram analysis. A hypnogram is defined as a diagram for identifying the sleep transition between different stages. These stages can be determined based on Rachtschaffen and Kales as wake, sleep with rapid eye movement (REM), non-REM stage 1 (NREM1), stage 2 (NREM2), stage 3 (NREM3), and stage 4 (NREM4) (Merica & Fortune, 2004). The hypnogram is generated from PSG signals in a period of 20 or 30 s epochs) (Rossow et al., 2011) as follows:

- Wake, comprising over half of the epoch, consists of alpha waves or low voltage, mixed-frequency (two–seven Hz) activity.
- Stage 1, comprising half of the epoch, consists of relatively low voltage, mixed-frequency (two–seven Hz) activity. At this stage, <50% of the epoch contains alpha activity. Slow rolling eye movements, lasting several seconds, can be often observed in early Stage 1.
- Stage 2 occurs with the appearance of sleep spindles and/or K complexes. Moreover, <20% of the epoch may contain high voltage (75 μV, <2 Hz) activity. Each sleep spindle and K complex have to last >0.5 s.
- Stage 3, comprising 20–50% of the epoch, consists of high voltage (>75 μV) and low-frequency (<2 Hz) activity.
- Stage 4, comprising over 50% of the epoch, consists of high voltage (>75 μV, <2 Hz) and delta activity.
- REM stage has a relatively low voltage that consists of mixed-frequency (two–seven Hz) electroencephalographic (EEG) activity with episodic REMs and absent or reduced chin electromyographic (EMG) activity (Maeda et al., 2007).

However, the main challenge in hypnogram analysis is the recognition of sleep stages, which is very time-consuming and, more importantly, depends on the analyst’s individual experience (Ronzhina et al., 2011; Gath & Bar-On, 1980). Hence, computerization of this process would be extremely helpful in saving time and in significantly enhancing the accuracy of sleep disorder diagnosis (Innocent, John & Garibaldi, 2004).

Many examples can be mentioned here regarding the application of intelligent techniques in medical diagnostic automation (Hassan & Haque, 2015a, 2016a, 2016b; Bashar, Hassan & Bhuiyan, 2015a; Hassan, 2015a, 2015b, 2016) and EEG analysis (Hassan & Haque, 2015b, 2015c, 2016a, 2017; Bashar, Hassan & Bhuiyan, 2015b; Hassan, Siuly & Zhang, 2016; Hassan & Subasi, 2016, 2017; Hassan & Bhuiyan, 2015, 2016a, 2016b, 2016c, 2017; Hassan, Bashar & Bhuiyan, 2015a, 2015b). In 2011, Kravoska et al. achieved 81% accuracy in sleep scoring using various features derived from PSG signals. In their work, they adopted a multidimensional analysis involving quadratic discriminant analysis. It was applied as a classifier using signal-specific features in different frequency bands (Krakovská & Mezeiová, 2011). Furthermore, in 2011, Kuo et al. used features based on multiscale permutation entropy in sleep scoring and achieved 89.1% sensitivity and over 70% accuracy in sleep scoring (Kuo & Liang, 2011). In another research by Hsu et al. (2013), multiple structures of artificial neural networks (ANNs) were applied based on energy-specific features from the signals. The obtained results indicated accuracies of 81.1%, 81.7%, and 87.2% for a feed-forward neural network, probabilistic neural network, and recurrent neural network, respectively. In 2016 (Hassan & Haque, 2016c), a combination of methods, based on complete ensemble empirical mode decomposition with adaptive noise (CEEMDAN) and bootstrap aggregating (bagging), was applied on PhysioNet data, which achieved 90.69% accuracy. In 2016, Hassan et al. worked on a single EEG for sleep scoring using normal inverse Gaussian parameters and achieved 90.01% accuracy (Hassan & Bhuiyan, 2017). Their other remarkable accomplishment was the achievement of 93.69% accuracy, which was obtained by using a tunable Q-wavelet transform (Hassan & Bhuiyan, 2016a).

Polysomnography analysis requires an optimal method for signal feature extraction. In this regard, wavelet tree decomposition can be particularly useful in extracting meaningful information from PSG signals for sleep scoring. Given the large amount of information generated by the wavelet tree analysis, it is necessary to reduce the dimension of data in a desirable way to make them usable for sleep scoring. In the present study, we introduced a step-by-step method for feature extraction using the wavelet tree analysis and dimensionality reduction using neighborhood component analysis (NCA). Moreover, we made a comparison between two well-known classifiers in sleep scoring, i.e., ANN and support vector machine (SVM).

## Methods

**Figure 1:**
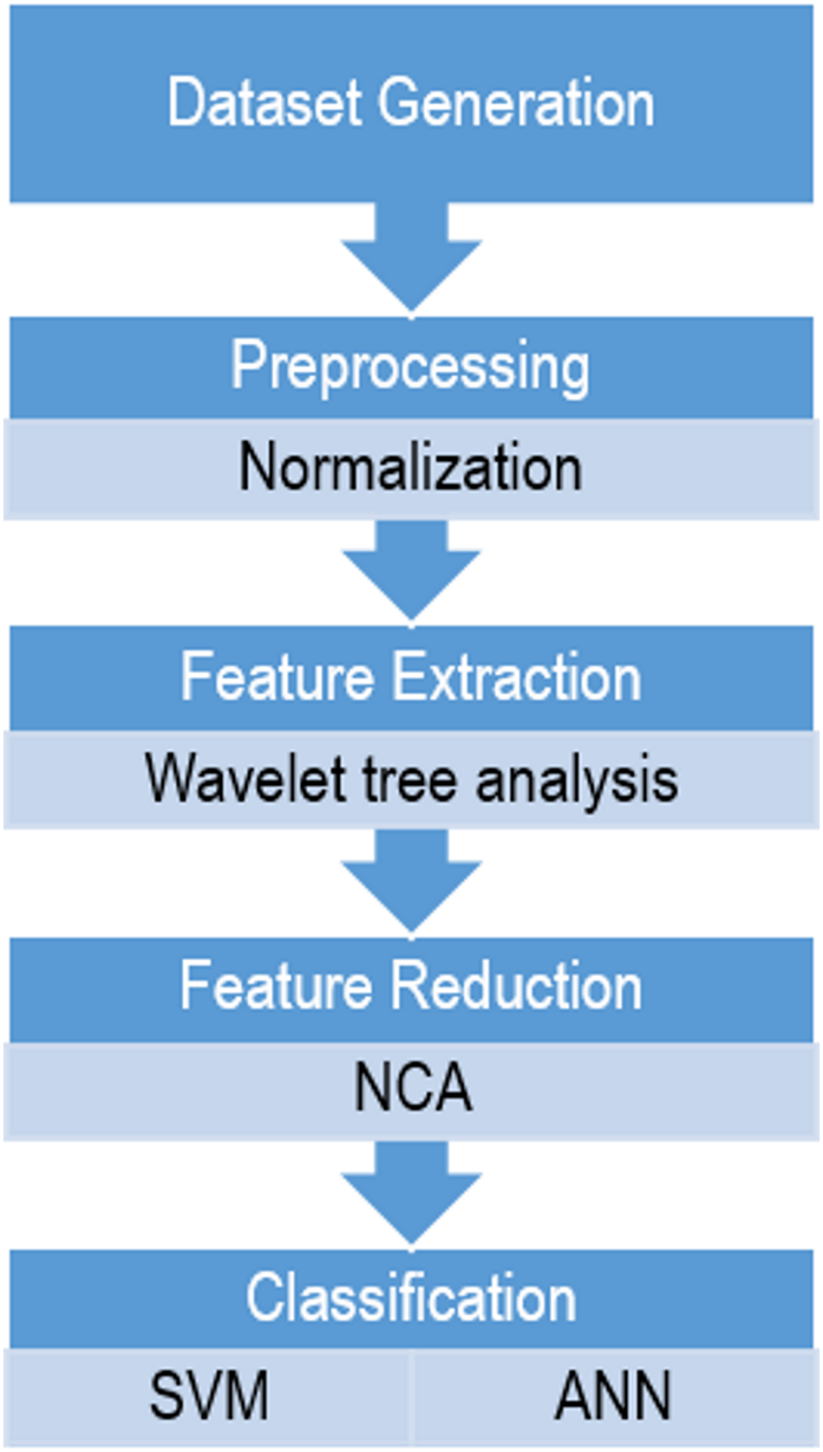
The flowchart of the proposed method for sleep scoring.

In order to compare these two classifiers based on wavelet features in sleep scoring, a sequential method was proposed in which the following steps were performed: dataset generation, preprocessing, feature extraction, dimensionality reduction, and classification, as shown in Fig. 1. All the steps were implemented using MATLAB 2016b (MathWorks, Natick, MA, USA).

### Data

**Figure 2:**
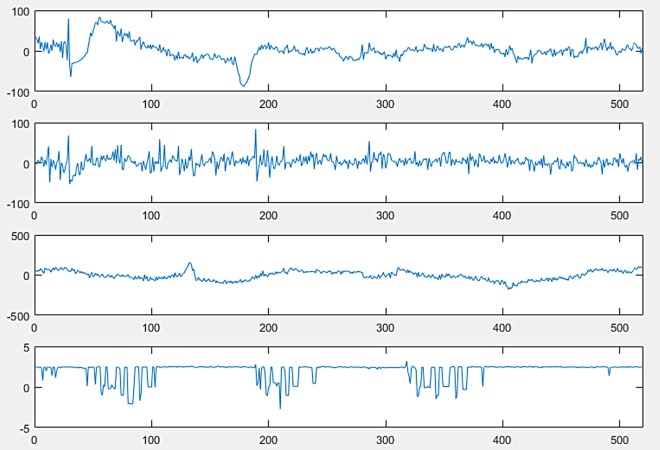
PolySomnoGraphy signal values.

The full version of sleep-EDF from PhysioNet, which is a collection of PSG recordings along with their annotated hypnograms, was used in this study as the initial dataset. The collection of 61 whole-night polysomnographic sleep recordings contained EEG signals of the Fpz-Cz and Pz-Oz channels, electrooculography (EOG) (horizontal), and submental chin EMG signals (Fig. 2) (Kemp, 2013). The EOG and EEG signals were sampled at 100 Hz. The submental EMG signal was electronically high-pass filtered, rectified, and low-pass filtered. Then, it was expressed in uV root-mean-square and sampled at one Hz (Kemp et al., 2000). In this dataset, hypnograms were generated for every 30 s of EEG data in accordance with the R&K criteria by well-trained experts (Hassan & Bhuiyan, 2016b).

A class-imbalanced dataset is one in which each class of the given dataset is not evenly distributed (Mohd Pozi et al., 2015). Notably, an imbalanced dataset is a serious problem in machine learning and data mining (Al Helal, Haydar & Mostafa, 2016). Because the number of sleep stages in the dataset was not equal (Table 1), 2,000 epochs were randomly selected from each sleep stage (Wake, REM, NREM1, NREM2, NREM3, and NREM4) and a 10,000-sample dataset was generated. It was actually done for the purpose of overcoming the imbalanced situation in the sleep-EDF dataset and reducing the next step’s computations. Although balancing the data can make a slight difference between the actual dataset and the new version, it does not make much sense as the number of samples was relatively high. In addition, balancing the dataset was necessary for classifier training in order to avoid biased learning.

**Table 1:** Stages count in sleep-EDF.

Stage	Count
Wake	77,327
N1	4,664
N2	26,560
N3	9,049
REM	11,618

### Preprocessing

In order to remove the noises from the signals, standard deviation normalization was applied as in Eq. (1). Actually, owing to the use of wavelet analysis in the next steps of the study, only standard deviation normalization was used to eliminate the noise in the first step. Further analysis of the noise reduction would be performed later using the wavelet transform.

(1) $${X_{{\rm{new}}}} = {{{X_{{\rm{old}}}}-{\rm{Mean}}} \over {{\rm{Std.dev}}}}$$

Equation (1). Standard deviation normalization.

This stage of preprocessing was performed to normalize the signals. Most of the noises were eliminated by multistage wavelet breakdown, owing to the use of the wavelet transform in the next step to extract the features.

### Feature extraction

**Figure 3:**
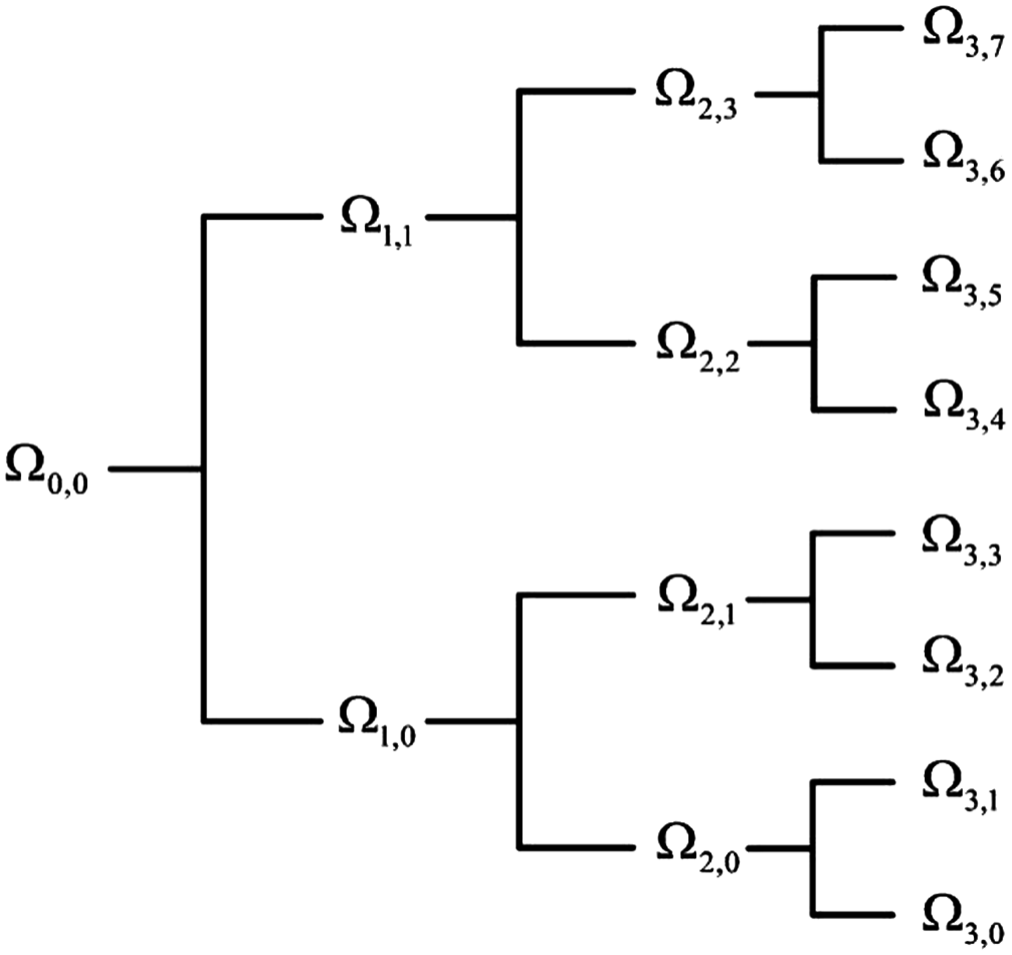
Wavelet packet feature extraction from input signal.

Considering the advancement of the wavelet transformation in analyzing non-stationary signals such as EEG, EOG, and EMG, the wavelet tree analysis was used for feature extraction in this step. Various features were generated based on the wavelet tree analysis (Khushaba et al., 2011; Savareh et al., 2017), which were used as the base features for sleep scoring. According to the wavelet feature extraction and the activity bands of input signals, a tree of wavelet decomposition was applied on signals at each level, and a group of features was generated (Fig. 3). Because it works based on multiresolution approximation by decomposing the signal into a lower resolution space (Aj) and details (Dj), the approximation space (low-frequency band) and detail space (high-frequency band) were frequently decomposed from the previous levels. This recursive splitting of vector space is represented by an admissible wavelet packet tree (Khushaba & Al-Jumaily, 2007). Energy was calculated using Eq. (2) for each subband of the signal.

(2) $$\log \left({S\left(l \right)} \right) = \log \left({\mathop \sum \limits_{m = 1}^\infty {{Wx{{\left({l,m} \right)}^2}} \over {Nl}}} \right)$$

*Wx* is the wavelet packet transform of signal; *l* is the subband frequency index; *Ni* is the number of wavelet coefficients in the *l*th subband.

Equation (2). Energy calculation of signals (Khushaba, Al-Jumaily & Al-Ani, 2007).

### Feature selection

Machine learning techniques require a suitable number of inputs to predict intended outputs in the most excellent way. Using a large number of inputs could affect the accuracy and lead to poor performance in many cases. This phenomenon is known as the curse of dimensionality, where increasing the number of features cannot guarantee performance improvement and may even lead to performance decay. Therefore, that phenomenon should be avoided as much as possible to maintain the classifier performance at a satisfactory level (Keogh & Mueen, 2011; Alizadeh Savareh et al., 2017).

In the present study, NCA was conducted to avoid the curse of dimensionality. In this technique, the importance of each input is calculated in the output prediction. Then, the important inputs are preserved for the next steps such as classification, fitting, and time series analysis. NCA learns a feature weighting vector by maximizing the expected leave-one-out (LOO) classification accuracy. NCA is a non-parametric method for selecting features with the goal of maximizing the prediction accuracy of the regression and classification algorithms (Yang, Wang & Zuo, 2012). Ideally, this algorithm aims to optimize the classifier performance in the future test data. However, because the real data distribution is not known, the algorithm attempts to optimize the performance based on the training data using the LOO mechanism. The algorithm is restricted to learning Mahalanobis (quadratic) distance metrics. It can always be represented by symmetric positive semi-definite matrices and it can estimate such metrics through its inverse square roots by learning a linear transform of the input space. If it is denoted by a transformation matrix A, a metric is effectively learned as *Q* = *A* > *A* in Eq. (3).

$$d\left({x,\;y} \right) = \;\left({x\;-\;y} \right) > Q\left({x\;-\;y} \right) = \;\left({Ax\;-\;Ay} \right) > \left({Ax\;-\;Ay} \right)$$ (3)

Equation (3). *Q* matrix calculation in NCA algorithm.

The goal of this algorithm is to maximize *f*(*A*), which is defined by Eq. (4), using a gradient-based optimizer such as delta-bar-delta or conjugate gradients.

(4) $$f\left(A \right) = \mathop \sum \limits_i \mathop \sum \limits_{j \in Ci} pij = \mathop \sum \limits_i pi$$

Equation (4). *f*(*A*): class separability as NCA maximization goal.

Because the cost function is not convex, some caution must be taken to avoid local maxima during training. Given the fact that its projection is linear, using a nonlinear classification is recommended in the core of the algorithm to avoid getting stuck in local maxima. This can be attained by using ANN and SVM, which are two well-known classifiers in machine learning techniques.

### Classification

A review of the literature shows that ANN and SVM have been used in other applications demonstrating the general acceptance of these techniques in different applications of classification tasks (Liu et al., 2018; Li et al., 2017). Therefore, in the present study, ANN and SVM, as the most popular and successful (Sammut & Webb, 2011) methods of machine learning, were also selected for sleep scoring.

### Artificial neural network

**Figure 4:**
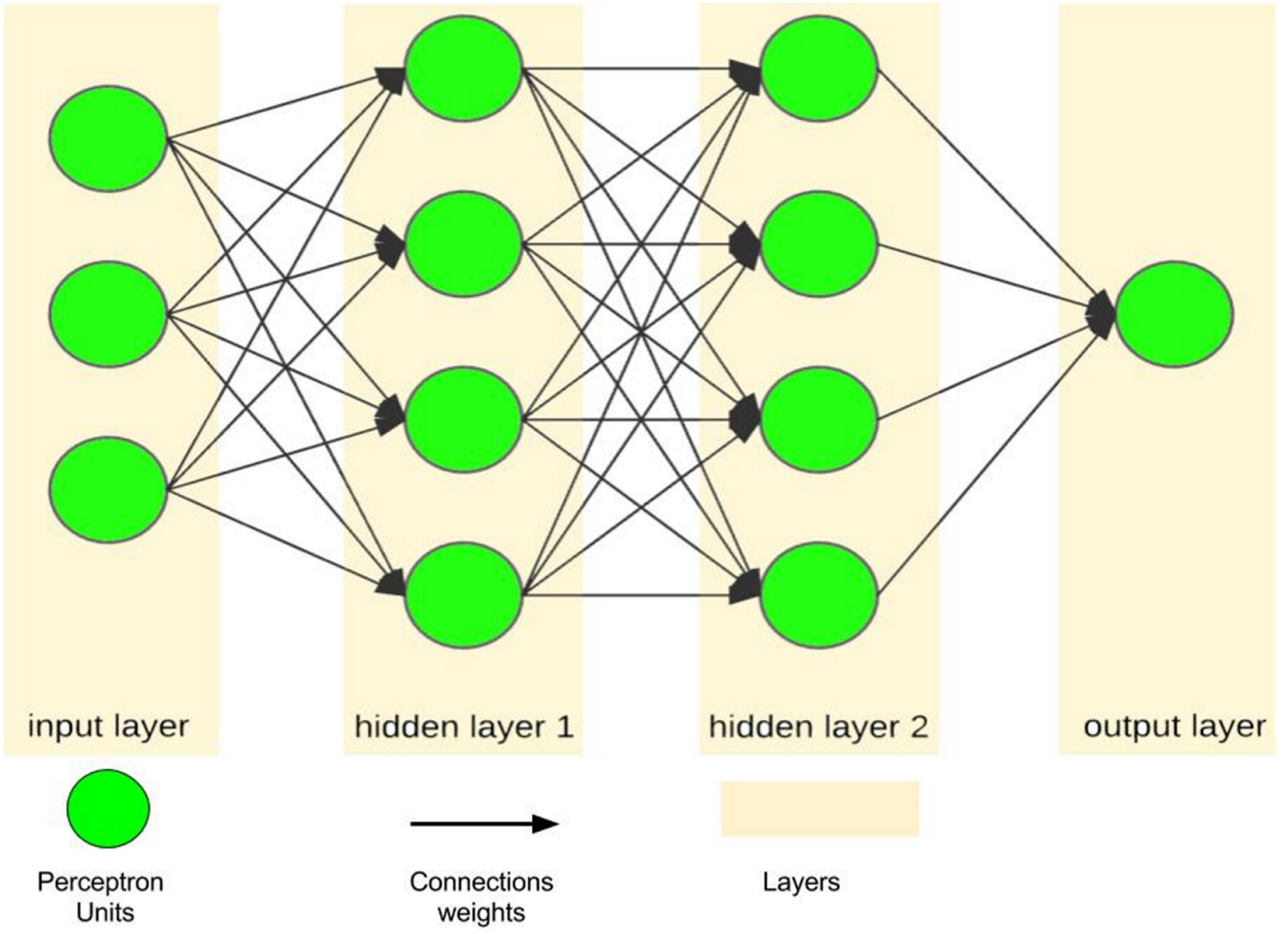
A sample of ANN with one input layer, two hidden layers and one output layer.

An artificial neural network, as a simple simulation of the human brain, tries to imitate the brain learning process using layers of processing units called perceptrons (Vaisla & Bhatt, 2010; Ferreira et al., 2008). A single perceptron, as the simplest feed-forward ANN unit, is only capable of learning a linear bi-class separation problem (Pradhan & Lee, 2010; Mohammadfam et al., 2015; Alizadeh et al., 2015). However, when a number of perceptrons are combined with each other in the layered structure, they emerge as a powerful mechanism with nonlinear separability called a multilayer perceptron, which is the most famous form of ANNs (Fig. 4). In this regard, ANN is considered as a logical structure with multiprocessing elements, which are connected through interlayer weights. The knowledge of ANN is presented through the weights adjusted during the learning steps. ANN is particularly valuable in processing situations where there is no linear or simple relation between inputs and outputs (Singh, Mahesh & Gupta, 2010) and in handling unstructured problems with data having no specific distribution models (Jani & Islam, 2012).

The main goal of ANN training is to reduce the error (*E*) of the classification as Eq. (5):

(5) $$E = {1 \over 2}\mathop \sum \limits_{i = 1}^m \mathop \sum \limits_{j = 1}^n {\left({yij-yi{j^{\rm{*}}}} \right)^2}$$

Equation (5). Error in ANN training phase.

In Eq. (5), *yij* and yij* are the actual and network outputs of the *j*th output from *i*th input vector respectively. In order to train and test the ANN structures, ANN models are implemented using the settings in Table 2.

**Table 2:** ANNs model setting in MATLAB.

Setting	Value
Activation function	Tangent sigmoid
Preprocess function	Remove constant rows
Data partitioning mode	Random
Network performance evaluation	Cross entropy
Iteration	1,000

### Support vector machine

**Figure 5:**
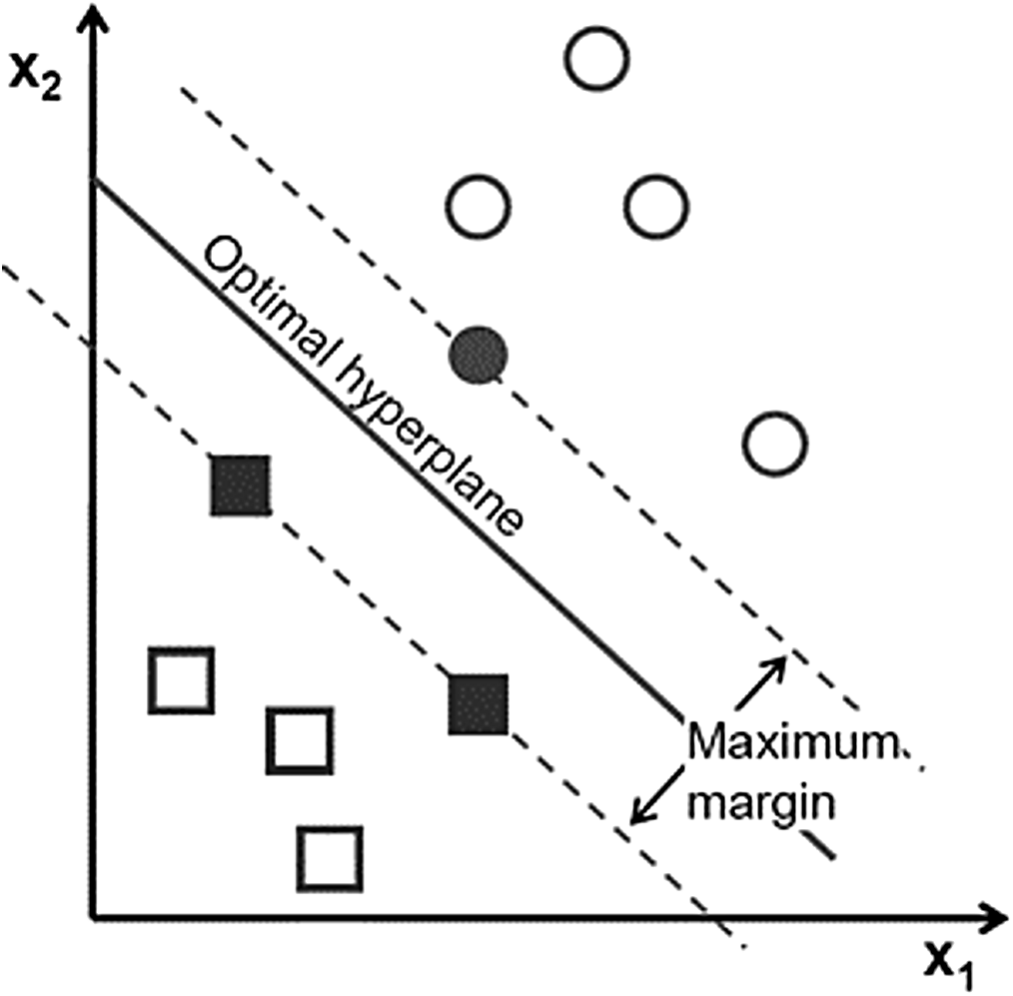
Support vector in SVM Each point shows a sample of data.

Support vector machine has become popular owing to its significantly better empirical performance compared with other techniques (Trivedi & Dey, 2013). SVM, with a strong mathematical basis, is closely related to some well-established theories in statistics and is capable of nonlinear separation using the hyperplane idea. It tries not only to correctly classify the training data, but also to maximize the margin for better generalization of the forthcoming data (Ge, Gao & Song, 2011). Its formulation leads to a separating hyperplane that depends only on the small fraction of data points lying on the classification margins called support vectors (bold texts in Fig. 5).

In the SVM training phase, tuning of the parameters involves choosing the kernel function and the box constraint (C). The box constraint is a tradeoff parameter between regularization and accuracy, which influences the behavior of support vector selection (De Leenheer & Aabi, 2001–2002). The kernel, as a key part of the SVM, is a function for transmitting information from the current space to a new hyperspace (Hsu, Chang & Lin, 2003). Because the Gaussian radial-basis function (RBF) kernel is popular, and RBF kernels are shown to perform better than linear or polynomial kernels (Bsoul, Minn & Tamil, 2011), the RBF function was selected in this study as the kernel for the SVM classifier. The RBF kernel is defined as Eq. (6), where σ is the most important factor to control the RBF kernel in transmitting data to a new hyperspace.

(6) $$K\left({x,x'} \right) = \exp \left({-{{x-{{x'}^2}} \over {2{{\rm{\sigma }}^2}}}} \right)$$

Equation (6). RBF kernel.

As mentioned earlier, to achieve the optimal performance, two parameters of SVM (box constraint (C) and RBF sigma (S)) are important and should be tuned as correctly as possible. To tune these parameters, two cycles are defined in terms of accuracy for exploring the values (Table 3) and choosing the best model with the highest accuracy.

**Table 3:** Parameters tuning.

Parameters	Setting
Gamma range	Outer product of log space (−1, 0.1, 10) and np.array([1, 10])
Box constraint range	Outer product of log space (−1, 0.1, 10) and np.array([1, 10])

### Validation of models

Validation of the results was performed in a different mode for each model. Intermittent ‘‘validation” was performed for ANN during training to avoid over-training problems. In this type of validation, the network is periodically validated with a different dataset. This process is repeated until the validation error begins to increase. At this point, ANN training is terminated, and the ANN is then tested with a third dataset to evaluate how effectively it has learned the generalized behavior (Omid, Mahmoudi & Omid, 2010). In this method, while training the network, as previously mentioned, 70% of the data were used to train the ANN whereas 15% were used for testing and 15% for validation purposes.

For the support vector, the cross-validation method was used to validate the modeling and testing. Cross-validation is a statistical method for evaluating and comparing learning algorithms. It is performed by dividing the data into two segments: one for learning or training the model and the other for validating the model. In a typical cross-validation, the training and validation sets must cross over in the successive rounds such that each data point has a chance of being validated. The basic form of cross-validation is *K*-fold cross-validation (Refaeilzadeh, Tang & Liu, 2009), which randomly divides the original sample into *K* subsamples. Then, a single subsample is selected as the validation data for testing the model, and the remaining *K*-1 subsamples are used as the training data. This process is repeated *K* times, and each *K* subsample is used exactly once as the validation data. The *K* results from the folds can then be averaged (or otherwise combined) to produce a single estimation (Jiang & Chen, 2016). This strategy was used for SVM validation using *K* = 10 and the mean accuracy was considered as the final accuracy for SVM.

## Results

Based on the activity bands of the input signals, six levels of wavelet tree feature extraction were used and a total number of approximately 3,500 features were generated for PSG signals in each epoch. As the large number of features can greatly increase the risk of the curse of dimensionality, the NCA algorithm was used for feature selection (to avoid the mentioned risk).

**Figure 6:**
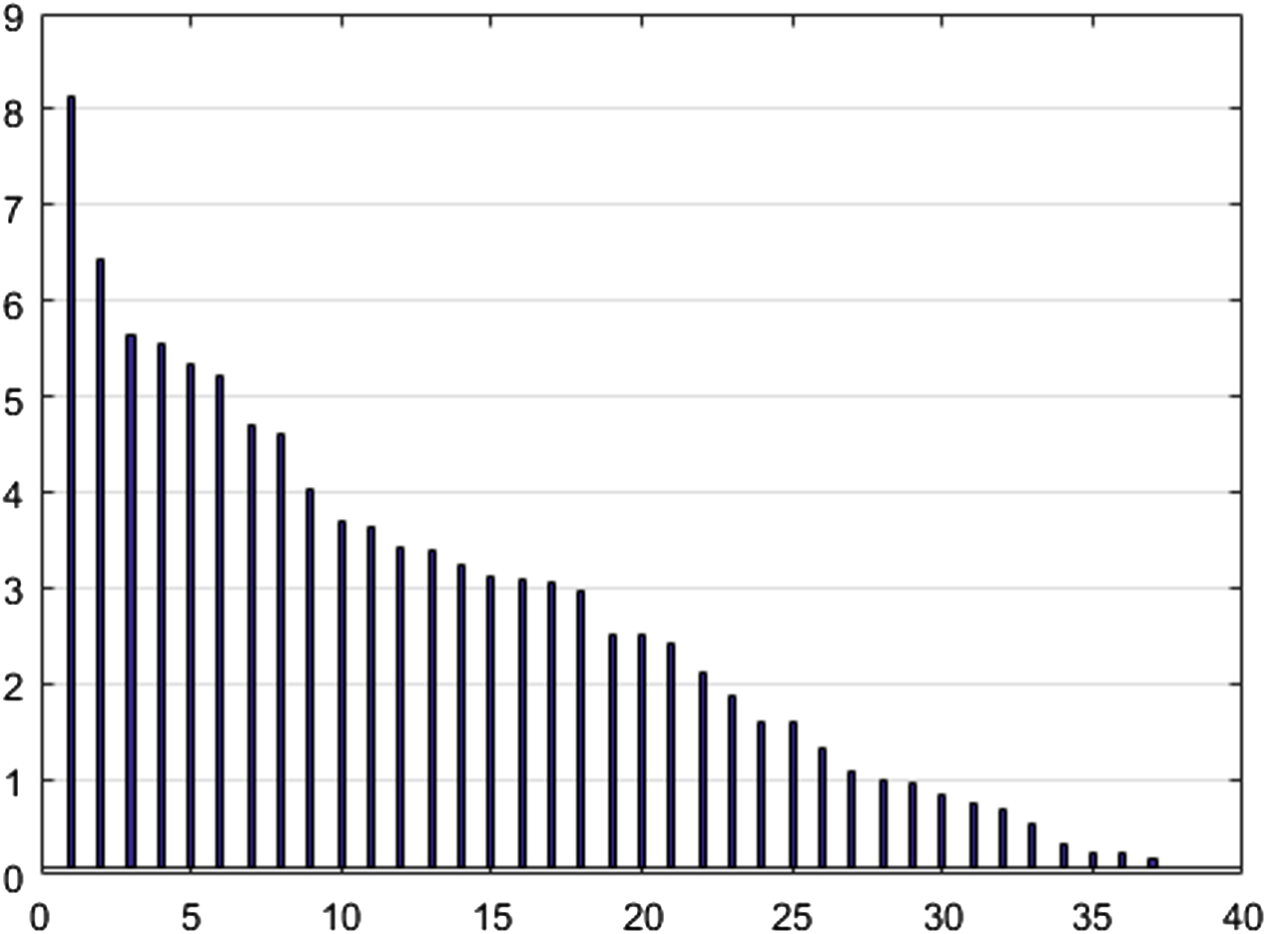
NCA output values.

To reduce the dimensions of the data using the NCA algorithm and to select the features, a threshold level of 0.1 was determined for weight screening. This value was selected by examining the appropriate number of output parameters based on threshold levels, where the goal of this step was to reduce the number of dimensions to 37. Figure 6 shows the NCA value (*y*-axis) for the selected features (*x*-axis) in a descending order.

**Figure 7:**
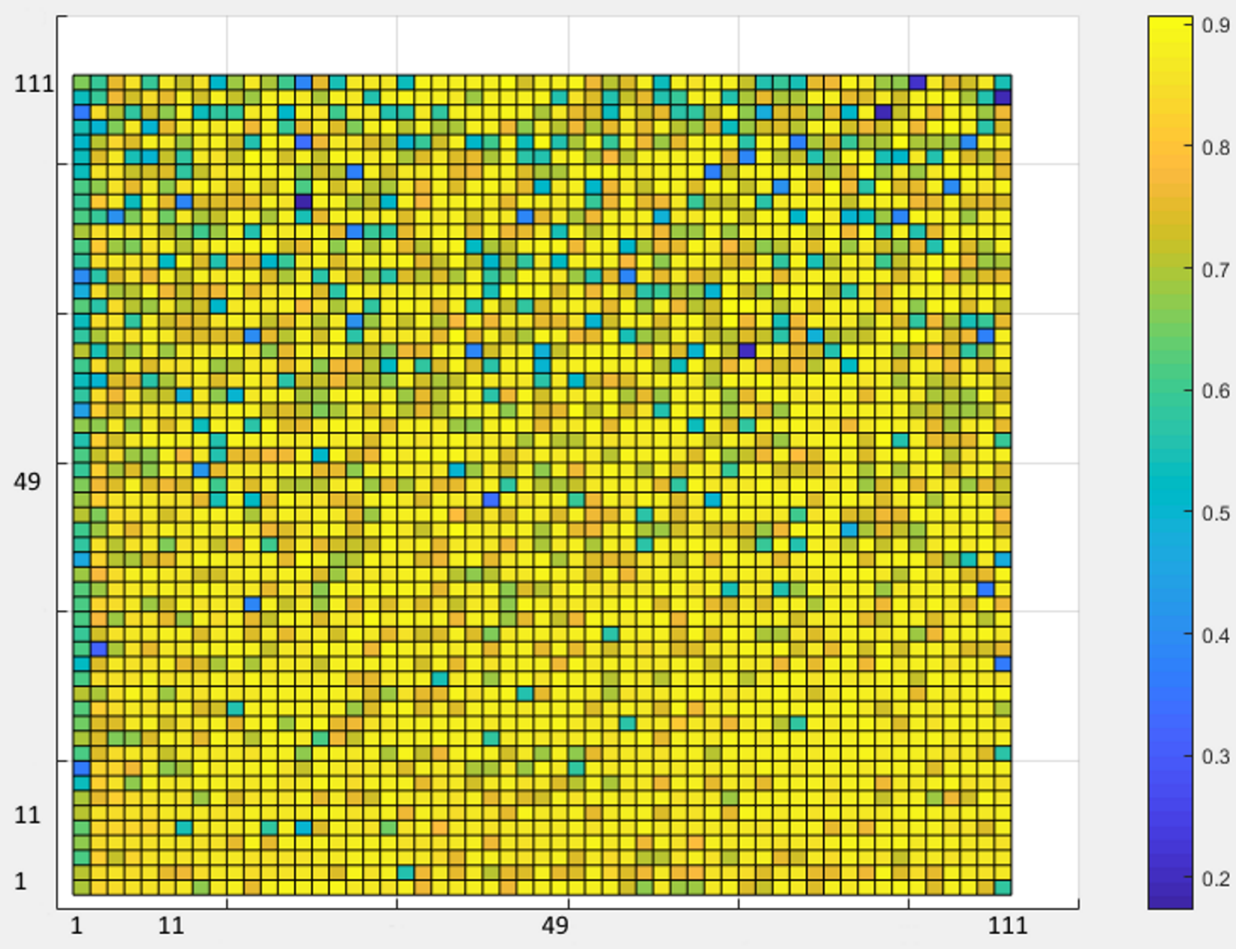
ANN Accuracy values.

As a rule of thumb, in the classification phase, all architectures with one or two hidden layers were investigated to achieve the best architecture in the ANN design. In each layer, as many neurons as one to three times the number of inputs were explored (Fig. 7).

**Figure 8:**
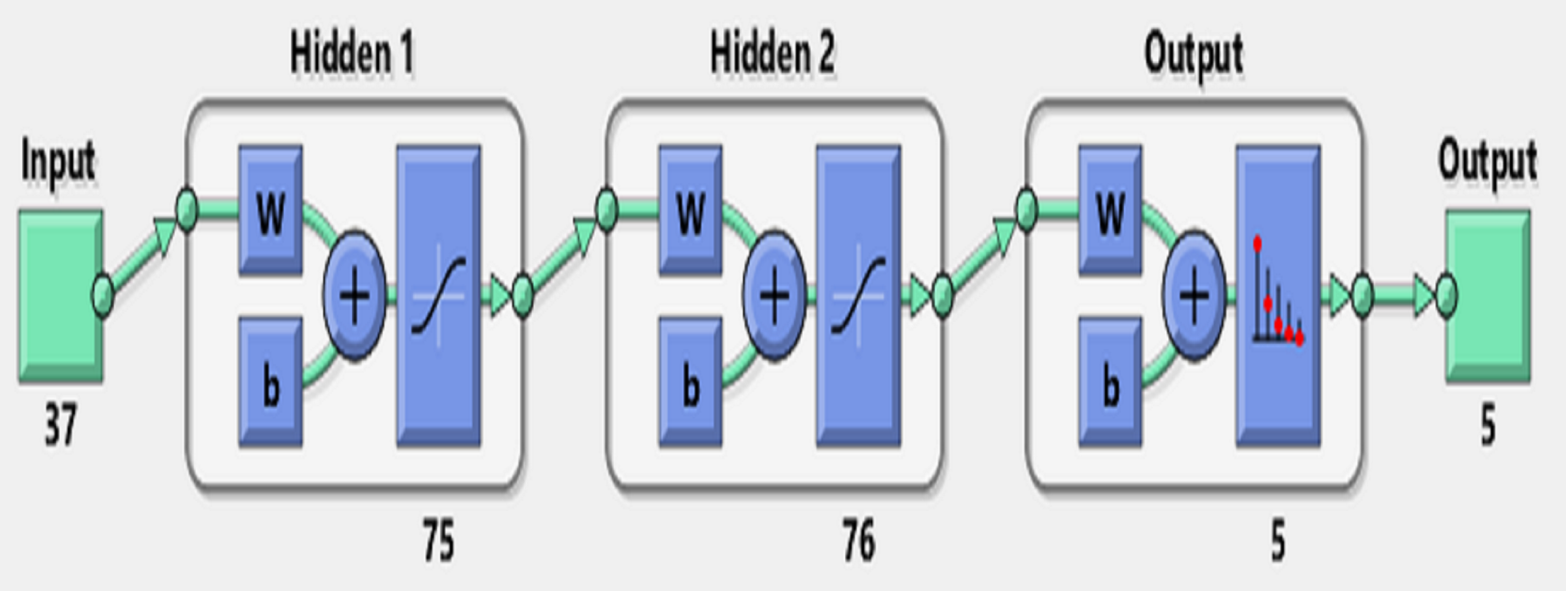
Artificial Neural Network Architecture for sleep scoring.

Figure 7 shows the accuracy values for different layering modes of the ANN, where the horizontal axis is the number of neurons in the first hidden layer and the vertical one is the number of neurons in the second hidden layer. Based on the results, an architecture with one input layer (37 neurons = number of selected features), two hidden layers (75 neurons, 76 neurons), and one output layer (with five neurons = the number of sleep stages) was considered as the optimal architecture (Fig. 8).

**Figure 9:**
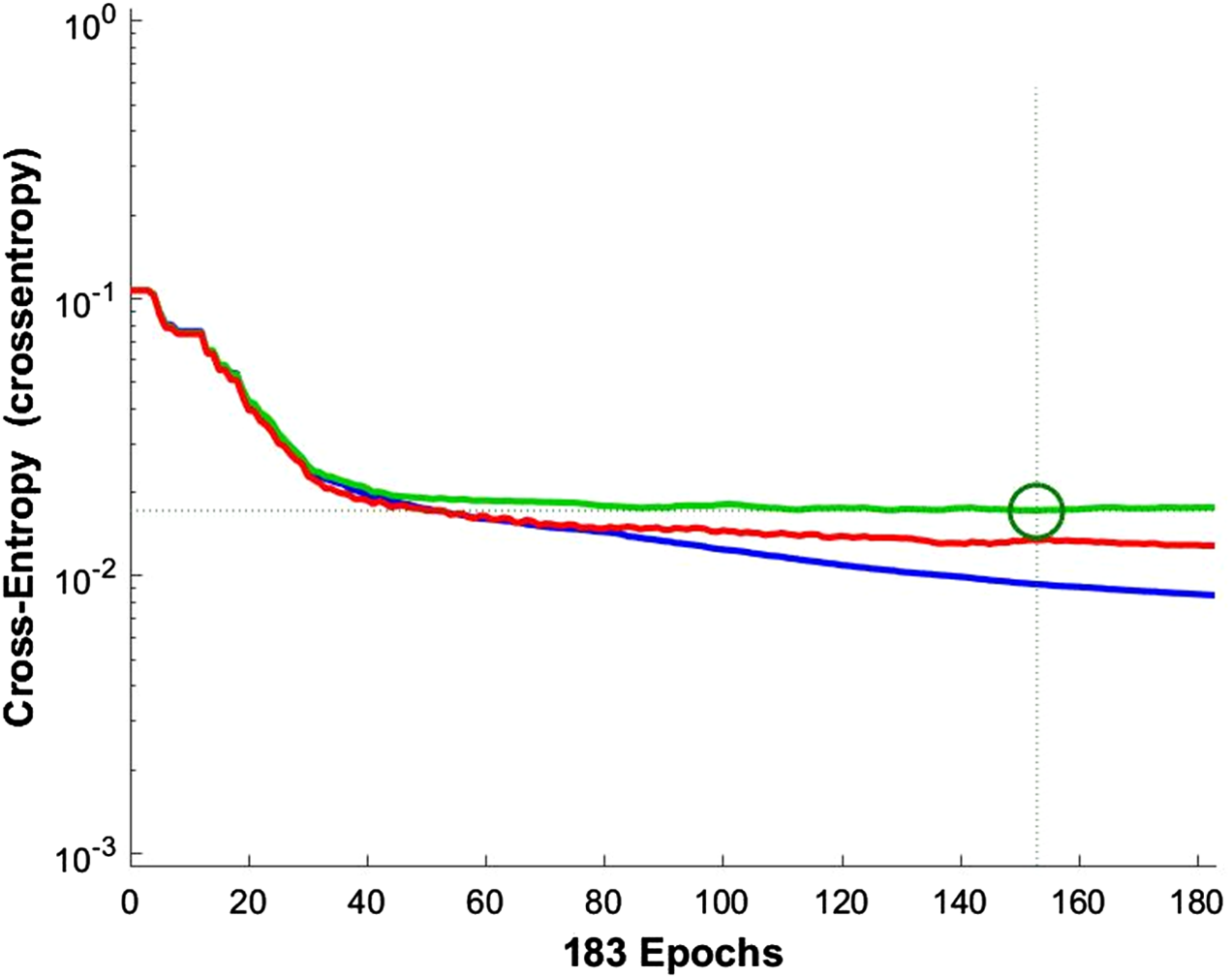
Network training cross entropy. The lines show the network performance. Blue, train; green, validation; red, test.

According to the information theory, if the target and predicted outputs of the ANN represent two probable distributions, their cross-entropy is a natural measure of their difference (Blumstein, Bitton & DaVeiga, 2006). It should be noted that cross-entropy is an appropriate criterion for assessing the training and controlling the ANN, if necessary. Figure 9 shows the cross-entropy values over epochs for network training.

**Figure 10:**
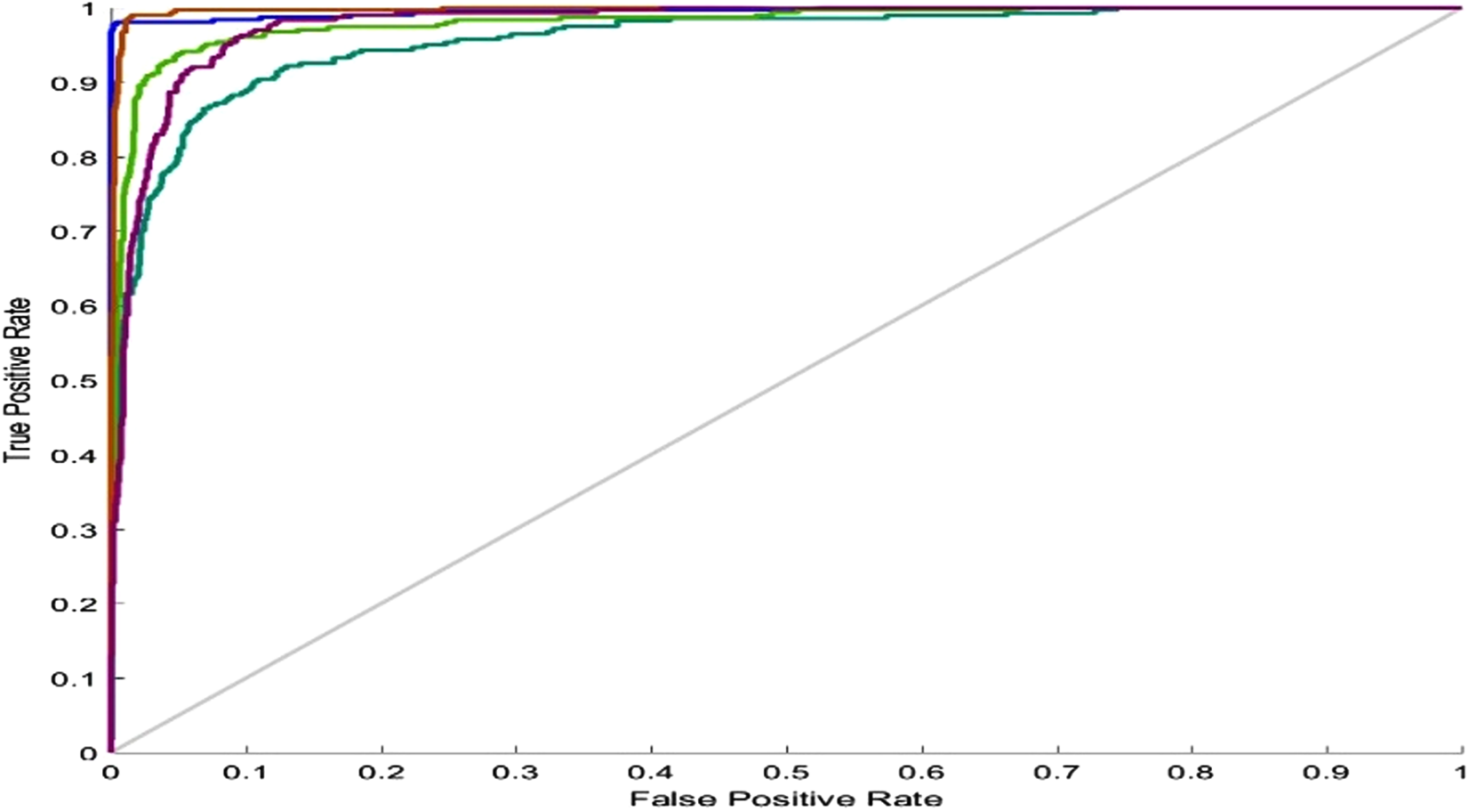
ANN ROC. ROC for five classes: blue, Wake; dark green, N1; light green, N2; red, N3; purple, REM.

For the five-class sleep scoring, ANN achieved a 90.3% accuracy, which is near the performance of the state-of-the-art method. As another assessment, the receiver operating characteristic (ROC) can be used as a statistic for the predictive test in a binary classification task. The ROC curve is a graphic representation of the sensitivity and specificity of the test across the entire range of the possible classification cut-offs. A 0.50 area under the ROC curve indicates a random test performance, whereas 1.00 is considered as perfect (Mattsson et al., 2012). Actually, these charts demonstrate the classifier’s ability to separate each class from the others. Converting the five-class classification problem into five binary classifications (each class versus the other classes) provides a benchmark for analyzing the classifier’s performance. Figure 10 shows the network performance on the test data section in the ROC curve.

**Figure 11:**
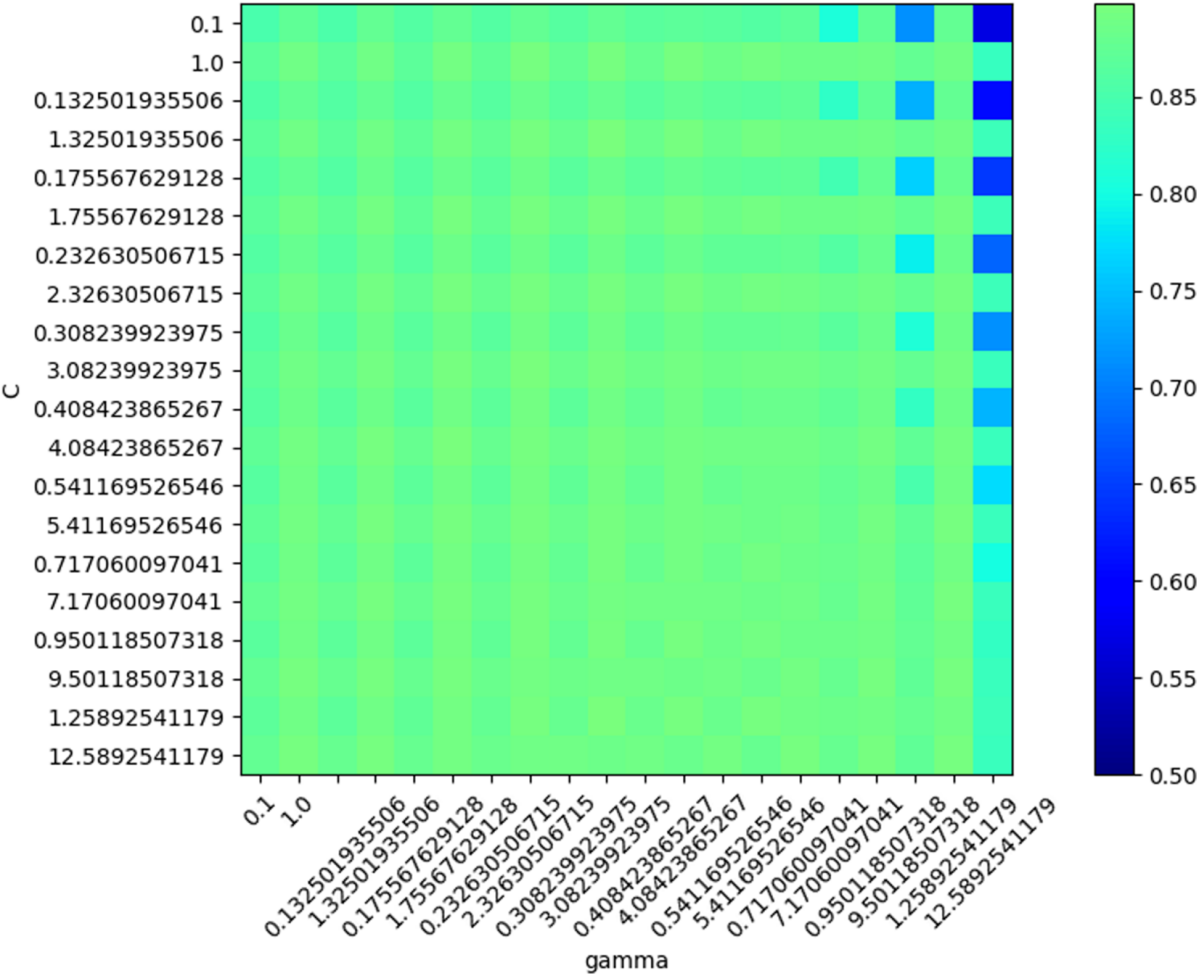
SVM Accuracy values.

In SVM training, various values were generated and tested as SVM parameters (box constraint and RBF sigma), and the accuracy was evaluated in each situation. The result of this step led to the creation of a chart of accuracy based on the parameters (Fig. 11).

**Figure 12:**
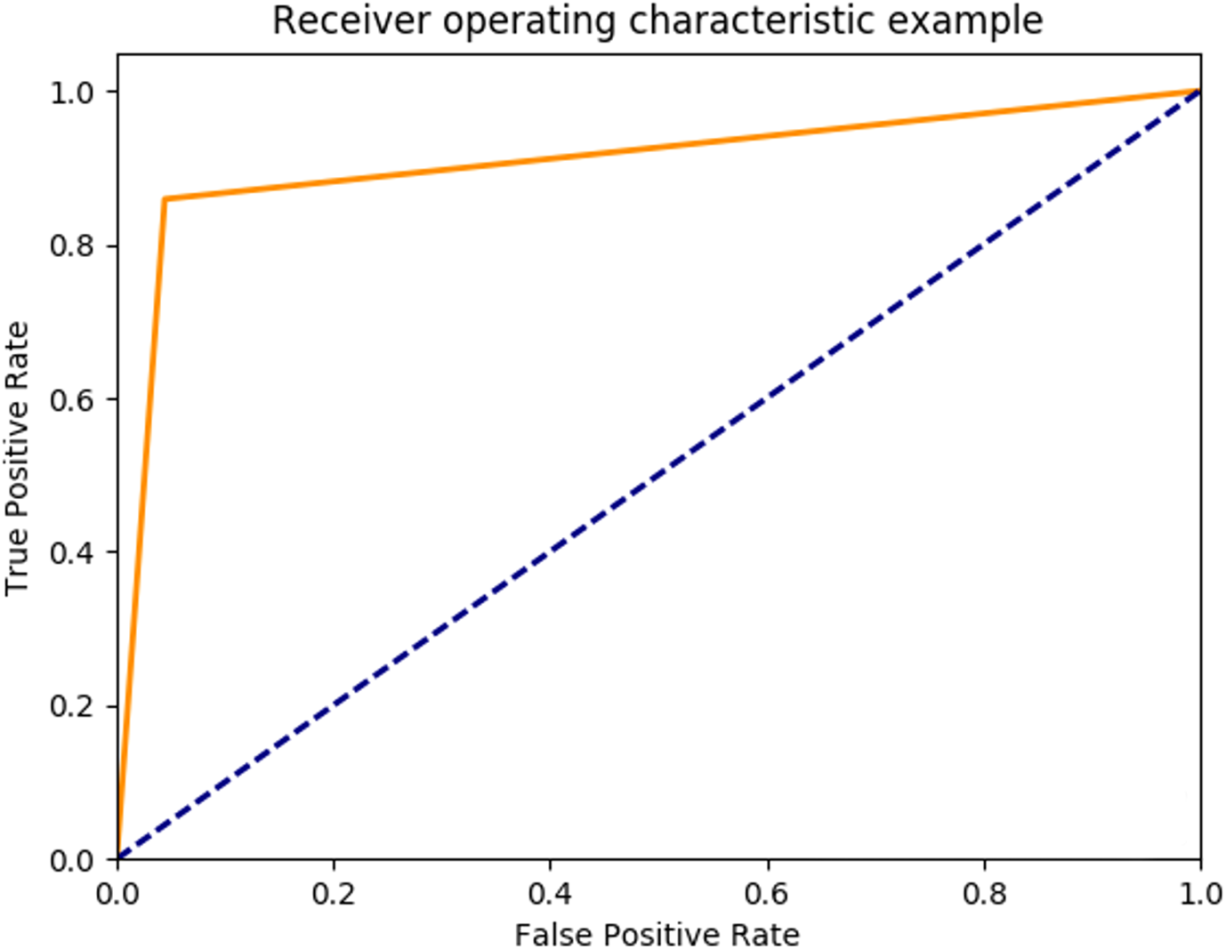
SVM ROC.

Based on the optimal parameters, the SVM model was created using the training samples, and a test was carried out based on the test samples. The SVM performance was evaluated as 89.93% in mean accuracy. Figure 12 shows the ROC diagram for SVM in a five-class sleep scoring with Area under the curve = 0.91.

**Figure 13:**
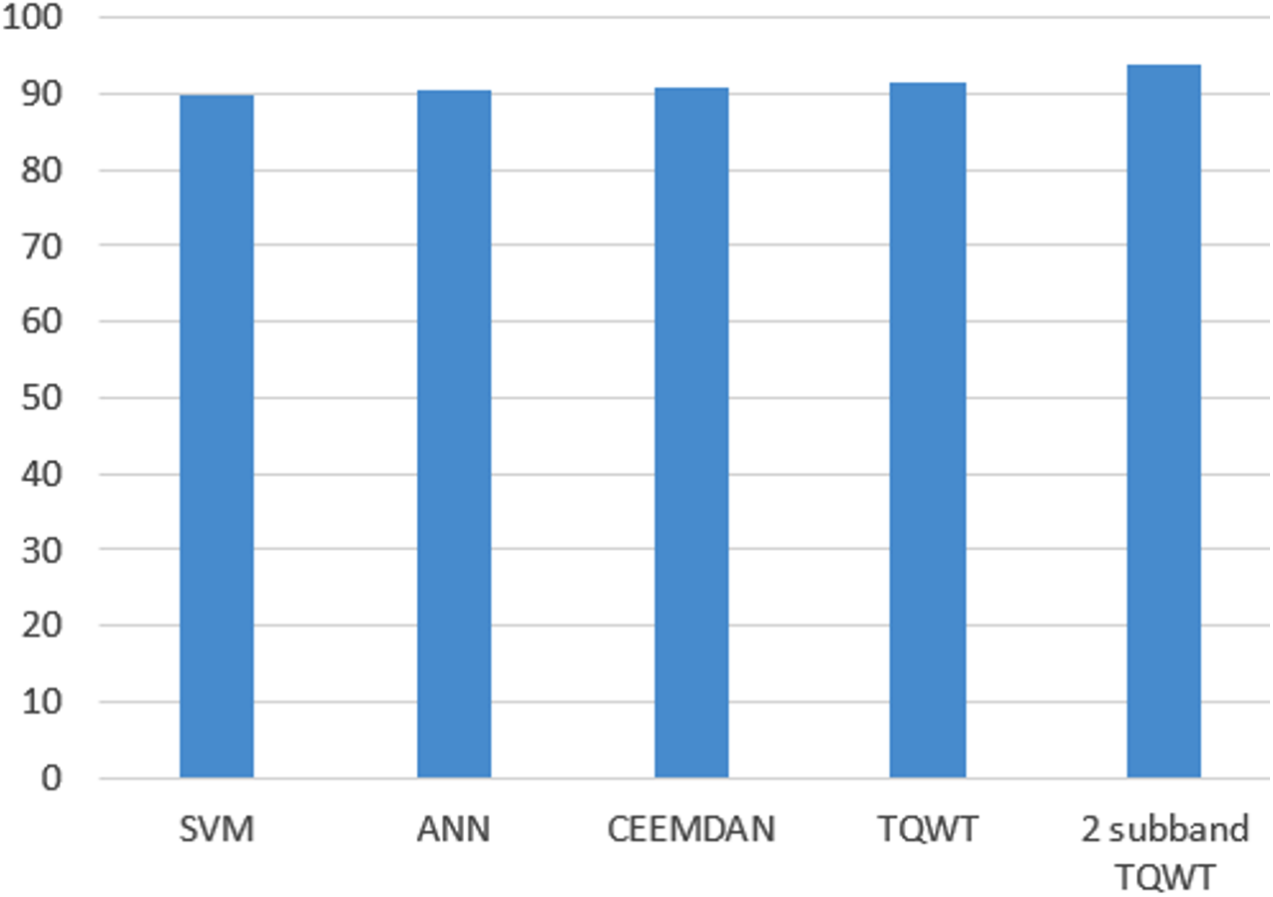
Accuracy comparison.

Furthermore, Fig. 13 shows a comparison of the performance of both ANN and SVM versus the state-of-the-art methods. As shown in the figure, the method introduced in this study achieved almost the same performance as that of the state of the art.

As stated in Maeda (2018), applying some primary criteria is important for evaluating the algorithms based on the validity of the reports. In the present study, the mentioned criteria were used as widely as possible in data preparation, data splitting, training the model, and reporting; however, each study, based on its intended purpose, examines a certain aspect of efficiency. Regarding the classification of sleep stages, choosing the accuracy as the main parameter of performance evaluation is an appropriate choice and has been considered in most sleep scoring studies. It should be noted that the cost of achieving the optimal performance was also examined for both ANN and SVM techniques. Given the different layers and nodes, the ANN training took a total of approximately 8 h on Intel Core i7 3 GHz laptop with 8 GB RAM, whereas checking different parameters of SVM took approximately 1 h on the same device.

## Discussion and Conclusion

The analysis of the studies on automatic sleep scoring reveals that the number of these studies is increasing in recent years (Hassan & Bhuiyan, 2015, 2016a, 2016b, 2016c, 2017; Hassan, Bashar & Bhuiyan, 2015a, 2015b; Hassan & Subasi, 2017). Moreover, the comparison of previous methods of sleep scoring with the introduced method in the present study showed some interesting points. In general, it can be concluded that the three phases including feature extraction, selection, and classification have been used in most of the studies.

In terms of features extracted from signals in the previous methods of sleep scoring, there were various techniques including spectral measures (Hassan & Bhuiyan, 2016a), nonlinear measures (Akgul et al., 2000), multiscale entropy (Liang et al., 2012), energy features from frequency bands (Hsu et al., 2013), and empirical mode decompositions (Hassan & Haque, 2016c). Moreover, features from dual tree complex wavelet transform, tunable Q-factor wavelet transform (Hassan & Bhuiyan, 2016a), normal inverse Gaussian pdf modeling (Hassan & Bhuiyan, 2017), and statistical moments (Hassan & Subasi, 2017) were used in the feature extraction phase.

The common property of these methods is the analysis of signal information at different times and frequency resolutions, which provide a detailed information of the signal at different levels.

Of course, the nature of biological signals, particularly those related to the brain function, show non-stationary properties and therefore, requires a combined time-frequency analysis simultaneously. It should be noted that the advantage of the method used in this study is the capability to perform simultaneous time-frequency analysis of the signals with high precision, and to finally present them in the form of energy parameters.

Energy extraction with the help of the multispectral analysis is valuable in the analysis of PSG signals. However, the volume of generated information is very high and each epoch of the PSG signals is mapped to a new sample in a space with a very high dimensionality. Therefore, it is necessary to control the huge amount of generated information to prevent the curse of dimensionality risk in the sleep scoring process.

In this regard, various methods have been used to reduce the dimension including manual selection of features, using transforms such as Quadratic and Linear discriminant analysis, and statistical analysis. In the present study, NCA, which combines linear and nonlinear analysis simultaneously, was used to reduce the number of dimensions. It decreases the dimensions based on a combination of linear and nonlinear operations in a mixed mode. According to the results from NCA, this method reduced the initial number of features generated by the wavelet tree analysis to 37 with a compression rate of approximately 0.01. In addition to the quantitative power of the method in compressing the feature dimensions, the selected features were also of excellent quality when they were used at the next stage as the input of the classifiers, leading to an acceptable performance.

Surveying studies have applied various classifier techniques such as Quadratic Discriminant Analysis (QDA), Linear Discriminant Analysis (LDA), ANNs, boosted decision tree, random forest, bagging (ANN), and adaptive boosting in sleep scoring. In this study, ANN and SVM were used for testing sleep scoring based on the features generated by the wavelet tree analysis. The features were then compressed using the NCA algorithm. One of the most successful studies in automatic sleep scoring applied CEEMDAN with bootstrap aggregating (bagging with a decision tree core) and achieved a 90.69% accuracy in sleep scoring (Hassan & Bhuiyan, 2016b). Another study applied tunable Q-wavelet transform features with various spectral features and achieved an overall accuracy of 91.50% for a five-class sleep scoring (Hassan & Bhuiyan, 2016a). Moreover, another study achieved 93.69% accuracy using a decomposed two-subband tunable Q-wavelet transform and four statistical moments extracted for each subband (Hassan & Subasi, 2017). In terms of overall accuracy (five-class separation), applying our methods on the sleep-EDF dataset achieved 90.33% and 89.93% accuracies for ANN and SVM respectively, which are close to the performance of the state of the art (see Tables 4–7).

**Table 4:** ANN confusion matrix.

Target/Out	Wake	N1	N2	N3	Rem
Wake	305	3	0	1	1
N1	5	256	6	0	8
N2	0	11	252	7	43
N3	0	1	6	277	0
Rem	5	22	6	20	265

**Table 5:** ANN evaluation metrics.

Metrics	Values
Accuracy	0.9033
Error	0.0967
Sensitivity	0.9057
Specificity	0.9758
Precision	0.9039
False positive rate	0.0242
F1_score	0.9034
Matthews correlation coefficient	0.8803
Kappa	0.6979

**Table 6:** SVM confusion matrix.

Target/Out	Wake	N1	N2	N3	Rem
Wake	292	5	0	0	1
N1	2	232	19	3	44
N2	0	5	275	10	7
N3	1	2	8	288	1
Rem	1	32	10	0	262

**Table 7:** SVM evaluation metrics.

Metrics	Values
Accuracy	0.8993
Error	0.1007
Sensitivity	0.8996
Specificity	0.9748
Precision	0.8994
False positive rate	0.0252
F1_score	0.8991
Matthews correlation coefficient	0.8743
Kappa	0.6854

In the end, the following points are worth mentioning. In the present study, the wavelet tree analysis was used for feature extraction from biological signals both in the time and frequency domains, because of its ability to mine very precise information about the signal energy. Notably, the wavelet tree produced high-dimensional features, which should be handled using a suitable method. In this regard, the NCA, as a combination of linear and nonlinear methods, was used to compress the information in an excellent way, both quantitatively and qualitatively. Thus, the advantage of this study was the use of the NCA method in reducing the dimensions of features appropriately by the simultaneous analysis of both linear and nonlinear features (although some similar studies had also achieved a good performance using some other classifiers). Given the modular capability of the method presented in this study, it is possible to replace any of its elements in the feature extraction, feature compression, and classification. Therefore, future studies can be directed toward changing each element to achieve better performance.

## Limitations

This study was limited to the acquisition of local sleep EEG datasets. Accessing such datasets could help validate its results more accurately.

## Supplemental Information

10.7717/peerj.5247/supp-1 Supplemental Information 1 ANN analyser.

10.7717/peerj.5247/supp-2 Supplemental Information 2 Load data.

10.7717/peerj.5247/supp-3 Supplemental Information 3 Master.

10.7717/peerj.5247/supp-4 Supplemental Information 4 Network.

10.7717/peerj.5247/supp-5 Supplemental Information 5 Report.

10.7717/peerj.5247/supp-6 Supplemental Information 6 SVM.

10.7717/peerj.5247/supp-7 Supplemental Information 7 SVM analyser.

## Additional Information and Declarations

### Data Availability

The following information was supplied regarding data availability: MATLAB code for the implementation of the methods are available as Supplemental Files.
